# Mechanisms of COVID-19-induced kidney injury and current pharmacotherapies

**DOI:** 10.1007/s00011-021-01520-8

**Published:** 2021-11-21

**Authors:** Wissam H. Faour, Ali Choaib, Elio Issa, Francesca El Choueiry, Khodor Shbaklo, Maryline Alhajj, Ramy Touma Sawaya, Zeina Harhous, Eman Alefishat, Moni Nader

**Affiliations:** 1 Gilbert and Rose-Marie Chagoury School of Medicine, Lebanese American University, P.O. Box 36, Byblos, Lebanon; 2 Department of Pharmacology, College of Medicine and Health Sciences, Khalifa University of Science and Technology, P.O. Box 127788, Abu Dhabi, United Arab Emirates; 3 Department of Physiology and Immunology, College of Medicine and Health Sciences, Khalifa University of Science and Technology, P.O. Box 127788, Abu Dhabi, United Arab Emirates; 4 Center for Biotechnology, Khalifa University of Science and Technology, Abu Dhabi, United Arab Emirates; 5 Department of Biopharmaceutics and Clinical Pharmacy, School of Pharmacy, The University of Jordan, Amman, Jordan

**Keywords:** COVID-19, Kidney, Renal pathology, Inflammation, Angiotensin

## Abstract

The COVID-19 pandemic created a worldwide debilitating health crisis with the entire humanity suffering from the deleterious effects associated with the high infectivity and mortality rates. While significant evidence is currently available online and targets various aspects of the disease, both inflammatory and noninflammatory kidney manifestations secondary to COVID-19 infection are still largely underrepresented. In this review, we summarized current knowledge about COVID-19-related kidney manifestations, their pathologic mechanisms as well as various pharmacotherapies used to treat patients with COVID-19. We also shed light on the effect of these medications on kidney functions that can further enhance renal damage secondary to the illness.

## Overview of the COVID-19 pandemic

Coronaviruses have caused two epidemics in the past 2 decades, the Severe Acute Respiratory Syndrome (SARS) and the Middle East Respiratory Syndrome (MERS) [1]. In December 2019, a novel coronavirus, later was named Severe Acute Respiratory Syndrome Coronavirus-2 (SARS-CoV-2), began to spread in Wuhan, China. It has then rapidly spread worldwide, and the World Health Organization (WHO) declared this outbreak a pandemic on the 11^t^^h^ of March, 2020 [2]. The WHO has officially named the infectious disease that is caused by SARS-CoV-2 as Coronavirus Disease-2019 (COVID-19) [3]. At the time of this writing, the WHO reported a little over 236 M confirmed COVID-19 cases and 4.8 M related deaths globally.

SARS-CoV-2 belongs to the Coronaviruses family; it shares 79.6% sequence identity with the previously identified SARS-CoV-1 [1, 4]. Studies done by Stockman et al., during the SARS outbreak in 2002–2003 revealed no significant improvement in patients treated with steroids but clear manifestations of side effects such as diabetes, avascular necrosis, psychosis, and prolonged viremia [5]. SARS-CoV-2 spreads majorly through droplets, aerosols, and direct contact, while it is detected in stool, urine, and blood [6, 7]. It enters the host cell through binding to angiotensin-converting enzyme II (ACE2) receptors that are abundant in the lungs, heart, blood vessels, and intestines [2]. Once in the cytoplasm, SARS-CoV-2 releases its genomic RNA and starts replicating inside the host cell [1]. Its median incubation period is estimated to be 5.1 days, with 97.5% of symptomatic infections becoming evident within 11.5 days [8].

Clinically, features of COVID-19 range from asymptomatic to acute respiratory distress syndrome (ARDS) and multi-organ dysfunction. The most common clinical features include coughing, fever, headache, sore throat, fatigue, and breathlessness. In some patients, the disease may adversely progress to pneumonia, respiratory failure, and death [9, 10]. This progression results basically from a severe inflammatory response characterized by an extreme rise of inflammatory cytokines and chemokines, which include IL-2, IL-7, IL-10, granulocyte colony-stimulating factor (GCSF), monocyte chemoattractant protein (MCP1), macrophage inflammatory protein 1 alpha (MIP1A), tumor necrosis factor (TNF), CXC-chemokine ligand 10 (CXCL-10), and C-reactive protein [11, 12]. Accumulating evidence suggest that the severity of COVID-19 is directly associated with increased levels of the above-listed cytokines and chemokines [12]. Noteworthy, among all the elevated inflammatory mediators, the blood IL-6 level is highly correlated with disease mortality, which suggests that fatal COVID-19 is characterized by a cytokine release syndrome (CRS) induced by a cytokine storm [13–15].

The kidney is among the different organs that are significantly afflicted by the SARS-CoV-2 infection. In this regard, studies have reported that many patients with COVID-19 pneumonia have presented multiple types of kidney injuries, while others who have died from COVID-19 illness showed severe kidney damage [16]. We will review herein the clinical manifestations of kidney injury in COVID-19 subjects with a focus on the currently approved treatment/vaccines and their effect on renal function.

## Clinical manifestations

Many reports have shown that renal dysfunction is an increasing clinical indicator of COVID-19 propagation. The most common clinical manifestation is proteinuria, which is found in more than half of the COVID-19 patients, in addition to hematuria, elevated blood urea nitrogen, and elevated serum creatinine. Moreover, radiographic abnormalities of the kidneys have also been observed [17–20]. In addition, SARS-CoV-2 was detected in urine analysis and postmortem samplings from the kidney tissues of the infected patients, confirming that the kidney is a definite target to these viral particles [21, 22]. From the pathological point of view, inflammation, edema, and a reduced density have also been reported in suffering kidney tissues [18]. Acute kidney injury (AKI) is infrequent in the context of mild-to-moderate COVID-19 individuals (5%). In these patients, the most common kidney abnormalities were subclinical [23]. Nevertheless, recent evidence shows that AKI is more common in critically ill COVID-19 patients [24]. The majority of COVID-19 patients (80%) have mild/moderate symptoms, while the remaining 20% develop severe/critical infections requiring oxygen supplementation and cardiopulmonary support [25]. The inflammatory response has been correlated with the severity of SARS-CoV-2 infection, exhibiting increased IL-6, IL-2R, IL-8, IL-10, TNF-α, and WBC counts, including the neutrophil-to-CD8^+^ T cell ratio [26, 27]. The following parameters were suggested to be implicated in the progression from mild/moderate to severe/critical conditions: IL-2R level > 793.5 U/mL, WBC > 9.5 × 10^9/L or neutrophil count > 7.305 × 10^9/L. Similarly, overproduction of IL-6 levels and reduction in CD8^+^ T cells were more pronounced among severe/critical patients [26]. Significant increases in IL-2, IL-7, IL-10, IP-10, MCP1, MIP1A, GCSF, and TNF-α were also recorded in severe/critical cases of ICU patients [9]. CCL17 levels were also considered as predictive markers for the differentiation of mild/moderate cases from severe/critical COVID-19 infections, with higher CCL17 levels in mild/moderate cases during early infection [28].

## Renal cellular entry of SARS-CoV-2 and cellular damage

Although the respiratory system is the major target of COVID‐19, reports indicated that kidney involvement is frequent and ranges from mild proteinuria to an advanced acute kidney injury (AKI). Proposed mechanisms of kidney injury in COVID-19 patients include complex processes with virus-mediated damage, cytokine storm, Angiotensin II pathway activation, dysregulation of complement, hypercoagulation, and microangiopathy [16, 29].

### Mechanisms of renal entry

SARS-CoV-2 mainly binds ACE2 proteins, which are expressed in kidneys on the brush border of the apical membrane of proximal tubules and to a lesser extent in podocytes. Thus, it could be hypothesized that the virus enters the arteriole and the glomerular capillaries and initially infects the glomerular endothelial cells. Consequently, podocytes are infected, and the virus enters the tubular fluid and binds to its receptors in proximal tubules, leading to acute tubular necrosis protein leakage in Bowman’s capsule, collapsing glomerulopathy, and mitochondrial impairment [29]. Initially, the virus gains access to the kidneys through the bloodstream, whereby many COVID-19 patients were reported to have SARS-CoV-2 RNAemia [9]. Although viremia in COVID-19 subjects remains a matter of debate, the virus was found in extracellular vesicles, allowing a systemic spread across the body and damage of various organs, particularly the kidneys [30–32]. Of concern is the ability of the SARS-CoV-2 to integrate its RNA within the genomic DNA of the host cells after reverse transcription [33]. This could possibly translate into the expression of viral proteins in kidney cells, a transformation that could lead to autoimmune disease.

**Fig. 1:**
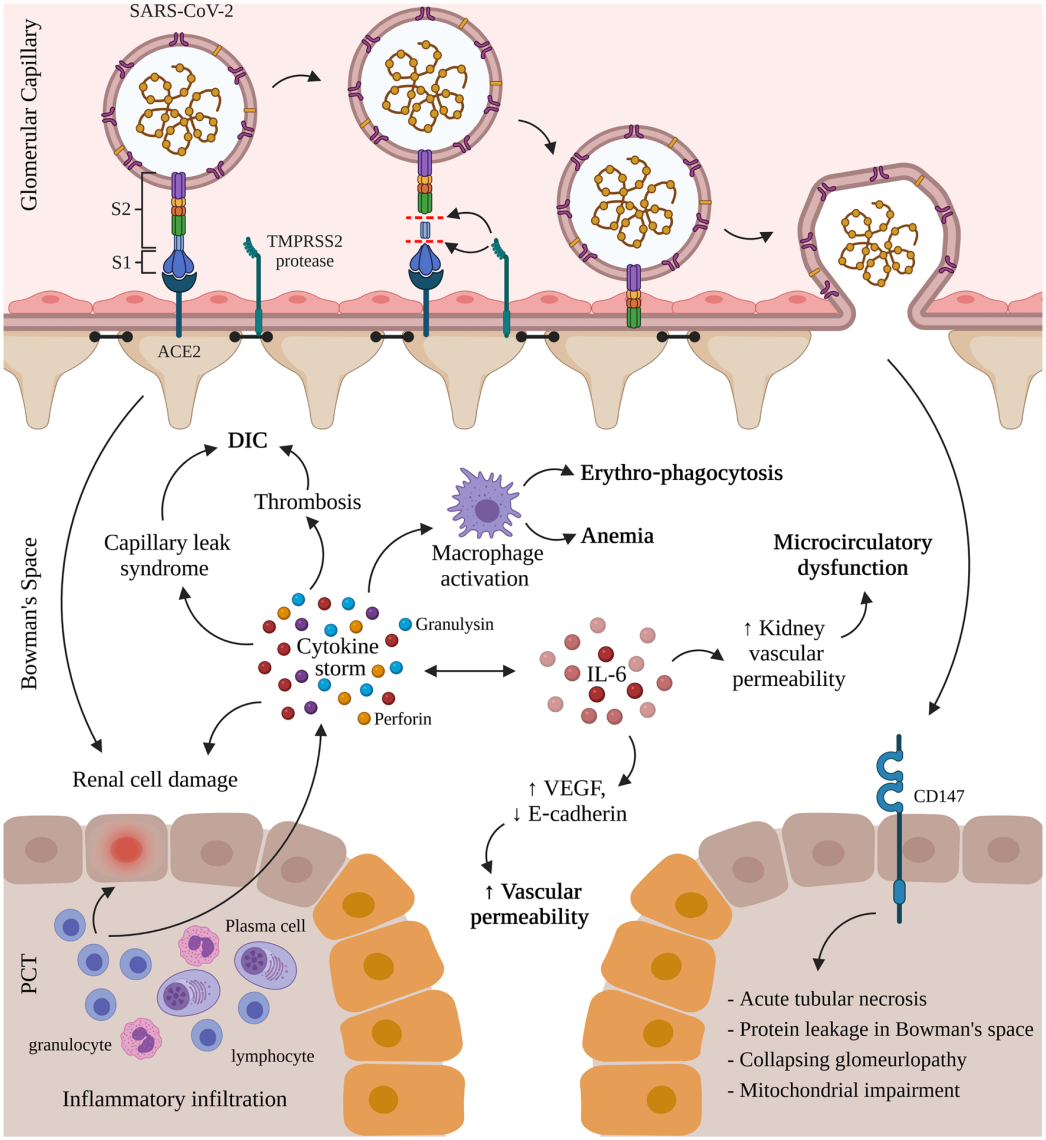
Mechanisms of renal entry and kidney injury in COVID-19 infection. COVID-19 infection in humans proceeds by the interaction of the receptor-binding domain (RBD) of the viral spike protein with the cell-surface angiotensin-converting enzyme II (ACE2). This is followed by the proteolytic cleavage of the spike protein through proteases like the transmembrane protease serine 2 (TMPRSS2). The virus interacts with CD147, expressed on the proximal convoluted tubules (PCT) of the nephron and on infiltrating inflammatory cells, resulting in acute tubular necrosis, protein leakage in Bowman’s capsule, collapsing glomerulopathy, and mitochondrial impairment. Simultaneously, the activated lymphocytes from the inflammatory infiltrates (lymphocytes, plasma cells and eosinophils) in the renal interstitium destroy renal cells and induce a cytokine storm of perforin, granulysin, and proinflammatory cytokines. The cytokine storm activates macrophages leading to erythro‐phagocytosis and anemia, induces capillary leak syndrome and thrombosis both linked to disseminated intravascular coagulation (DIC), and contributes to renal cell damage also caused by direct renal infection. Oversecretion of key cytokine, interleukin-6 (IL‐6), that binds the IL‐6 receptor and activates the vascular endothelial growth factor (VEGF), decreases the expression of E‐cadherin, increases vascular permeability, shock, and MOD while increasing kidney vascular permeability and microcirculatory dysfunction. (Created with Biorender.com)

Thus, the first step of SARS-CoV-2 infection in humans is the contact of the virus with cell-surface ACE2. ACE2 interacts with external SARS-CoV-2 by binding to the receptor-binding domain (RBD) of the viral spike protein. This process is followed by proteolytic cleavage of the spike protein, which allows fusion to cells, and transmembrane protease serine 2 (TMPRSS2) has been identified as a protease responsible for the reaction (Fig. 1).

Another recently studied mode of entry of the virus is through employing the NRP-1 receptor [34]. NRP-1 is a catalytic and signaling protein widely known for its role in cellular signaling and its function as a cell-surface receptor. It was shown to serve as an entry factor and to potentiate SARS-CoV-2 infectivity in vitro [34, 35]. NRP-1 has two isoforms: a truncated secreted form and a transmembrane form which interacts mainly with SARS-CoV-2 particles [34]. The transmembrane form has a ligand-binding site for growth factors such as VEGF, also co-opted by different viruses including EBV, human T cell lymphotropic virus-1 (HTLV-1) as well as SARS-CoV-2 [34–37]. In severe COVID-19 cases, arterial injury results in a potential upregulation of the NRP-1 receptor [32, 34]. Analysis of a cryopreserved diabetic kidney single-nucleus RNA sequencing dataset showed that NRP-1 was the only receptor significantly upregulated [34].

### Acute kidney injury in COVID-19 patients

According to a study done in China in 2020, 75.4% of 333 patients with SARS-CoV-2 infection had renal involvement, with proteinuria and hematuria. The incidence of AKI was estimated to be 4.7% in the total cohort by KDIGO criteria. Compared to those with moderate disease, a greater incidence of proteinuria and hematuria were demonstrated in patients with severe or critically ill COVID-19 pneumonia. In general, 43.9% of critically ill cases developed acute kidney injury during the hospital stay. The patients with renal involvement, including hematuria, proteinuria, and acute kidney injury, had a higher overall mortality of 11.2% compared to 1.2% of those without renal involvement [19]. Similarly, of 710 patients admitted to Wuhan Jin Yin-tan hospital with confirmed SARS-CoV-2 pneumonia, 52 patients were considered critically ill patients. Most patients had organ function damage, including 29% with acute kidney injury, 17% of those required renal replacement therapy [38]. Moreover, and in a different study, serum creatinine and blood urea nitrogen (BUN) were shown to be elevated while the glomerular filtration rate was low on the admission of patients with SARS-CoV-2 infection. In general, 3.9% of those patients had proteinuria, and 26.7% had hematuria. Interestingly, patients with elevated baseline serum creatinine demonstrated a higher leukocyte count and lower lymphocyte and platelet counts with coagulation pathway abnormalities linking to a critical disease. During hospitalization, acute kidney injury occurred in 5.1% of those patients with the incidence being highest in the subjects with elevated baseline serum creatinine. In-hospital death occurred in 33.7% of patients with elevated baseline serum creatinine, elevated baseline blood urea nitrogen, proteinuria, hematuria, and acute kidney injury [17]. In another study, acute kidney injury occurred in 46% of patients admitted at the Mount Sinai Health System during 2020 with COVID-19; 19% of those patients required dialysis. Although it seemed consistent, the proportions of patients with acute kidney injury and those in the ICU varied in the five Mount Sinai Health System locations. This study concluded that of the 4,000 patients with COVID-19 admitted to Mount Sinai Health System, acute kidney injury occurred in nearly half of the patients, and nearly a quarter of those patients required acute dialysis. Acute kidney injury was independently associated with higher mortality: 35% of survivors did not recover kidney function by hospital discharge and, of all patients with acute kidney injury, only 30% survived and regained kidney function [39]. Compared to previous pandemics with the same virus family, the current COVID-19 pandemic, just like the severe acute respiratory syndrome coronavirus 1 outbreak in 2005, yielded a higher incidence of AKI, opening thereafter a way for potential future research [40]. In a recent Brazilian cohort study, 55% of the patients developed AKI, and more than half of that progressed into stage 3 that was correlated with a high mortality rate compared to those without AKI [41]

In summary, while comparing different populations of COVID-19 patients in different locations and studies, it was shown that, clinically, patients with severe and critical COVID-19 infections were at substantially higher risk of developing acute kidney injury with varying outcomes of sustained kidney injury, recovery, need for renal replacement therapy, or death. Predictor factors for severe acute kidney injury overlapped between the different studies and included old age, high creatinine, and blood urea nitrogen at presentation, gender (being male), and history of hypertension and diabetes.

### Renin–angiotensin–aldosterone system impairment by SARS-CoV-2

The renin–angiotensin–aldosterone system (RAAS) regulates tissue perfusion, extracellular volume, and blood pressure homeostasis via opposing pressor and depressor pathways [42, 43]. Renin release is the first biochemical-limiting step in the activation of RAAS. The factors that contribute to the release of renin include reduced-sodium delivery to the distal convoluted tubule, reduced perfusion pressure in the afferent arteriole of the kidneys, hypovolemia, and sympathetic stimulation [44–46]. On the contrary, the release of renin is inhibited by the atrial natriuretic peptide (ANP), released secondary to the mechanical stretch of myocardial walls induced by volume overload or high blood pressure [47–49]. In the circulation, renin metabolizes angiotensinogen, liberating angiotensin I (Ang 1–10). Next, the angiotensin-converting enzyme (ACE), released from vascular endothelial cells in the lungs and in smaller proportions by the kidneys, converts Ang I (Ang 1–10) to the potent vasoconstrictor Angiotensin II (Ang 1–8). Ang II (Ang 1–8) acts to separately activate two G-protein coupled receptor subtypes, angiotensin type‐1 and type‐2 receptors (AT_1_R and AT_2_R, respectively), each of which mediates different physiological outcomes. Ang II (Ang 1–8)-mediated activation of AT_1_R in the endothelium of arterioles causes significant vasoconstriction, inflammation, and fibrotic remodeling, as well as sodium retention with aldosterone and renin release [44–46, 50]. Conversely, activation of AT_2_R by Ang II (Ang 1–8) initiates opposing effects to AT_1_R, hence producing vasodilatory effects and inhibiting growth [50]. On the other hand, ACE2 cleaves Ang I (1–10) into Ang 1–9 and converts Ang II (Ang 1–8) to Ang 1–7. Products of the ACE2 peptidase, Ang 1–9 and Ang 1–7 provide vasoprotection through vasodilatory, anti-inflammatory, and anti-fibrotic properties [50].

Under normal conditions, the two opposing pathways of the RAAS system coordinate to match increases in ACE and Ang II (pressor pathway) with rises in ACE2 and Ang 1–7 (depressor pathway) to keep homeostatic balance [51–53]. However, under pathological conditions, an imbalance between both pathways can be noted and is unopposed skewed towards ACE and Ang II activities, potentially driving renal injury [54, 55]. Similarly, upregulated levels of Ang II were found among COVID-19 patients [56], which raises the possibility of Ang II involvement in renal pathology. For example, the ion channel TRPC6 is directly activated in podocytes by Ang II and causes excessive proteinuria and kidney damage [57–59]. TRPC6 is further involved in manifestations of COVID-19 infection, including pain, and currently, a specific TRPC6 inhibitor is being developed by Boehringer Ingelheim. TRPC6 is heavily involved in pulmonary edema [60] and endothelial barrier dysfunction with increased endothelial permeability of pulmonary blood vessels [61].

During COVID-19 infection, the binding of the SARS-CoV-2 virus to its ACE2 receptor downregulates the latter and contributes to the loss of ACE2 catalytic activity in RAAS [62]. ACE2 loss promotes the activation of AT_1_R by AT_2_R, hence amplifies the oxidative stress response and cytokine production, contributing to more inflammation and disruption of the glomerular filtration barrier [63]. Studies on ACE2-deficient mice reported cardiovascular defects, including endothelial disruption [64], high blood pressure [65], and cardiac structural abnormalities [66]. Furthermore, a subsequent drop in Ang (1–7) from ACE2 inactivation might drive a shift towards the pressor pathway of RAAS and the corresponding deleterious cardiovascular effects in COVID-19 patients [50].

### Mechanisms of renal injury

COVID-19 causes kidney injury by either direct infection or systemic effects, including host immune clearance and immune tolerance disorders, endothelium-mediated vasculitis, thrombus formation, glucose and lipid metabolism disorder, and hypoxia [20]. Starting with direct renal infection, evidence suggested that SARS-CoV-2 binds to ACE2 through the S1 subunit, thus directly causing damage to intrinsic renal cells. Human tissue single-cell RNA sequencing data and ACE2 staining revealed that the kidneys and bladder are enriched with ACE2, which increases their chance of viral invasion [67, 68].

At the immunologic level, inflammatory infiltration of the renal interstitium predominantly consists of lymphocytes and plasma cells, with some eosinophils [69]. The activated lymphocytes migrate to kidney tissues to destroy infected renal cells and release inflammatory cytokines, which results in local inflammation and tissue injury. In addition, cytotoxic particles such as perforin, granulysin, and proinflammatory cytokines, which are highly expressed in lymphocytes, also contribute to kidney damage [70, 71]. The exaggerated release of cytokines leads to a cytokine storm [72, 73]. The cytokine storm may contribute to AKI in COVID‐19 cases by cooperating with renal resident cells and promoting tubular and endothelial dysfunction [29]. Among all cytokines, IL-6 has been a key player by inciting renal endothelial cells to secret pro‐inflammatory chemokines/cytokines and induced kidney vascular permeability, playing a part in microcirculatory dysfunction [72]. Pro‐inflammatory cytokines can also induce capillary leak syndrome and the production of thrombosis, which may result in disseminated intravascular coagulation [74]. Erythro‐phagocytosis and anemia are also observed since cytokines can activate macrophages (Fig. 1). Altogether, the disturbances of vascular hemostasis, anemia, and cytokine‐induced injuries lead to kidney failure [75].

The aforementioned conditions, as well as COVID‐19, may result in over secretion of IL‐6 that can bind to IL‐6 receptors leading to activation of vascular endothelial growth factor and decreased expression of E‐cadherin, promoting vascular permeability, shock, and MOD [76]. Mechanisms of kidney injury in COVID-19 infection are depicted in Fig. 1.

### Histopathologic findings (autopsies and biopsies)

**Fig. 2:**
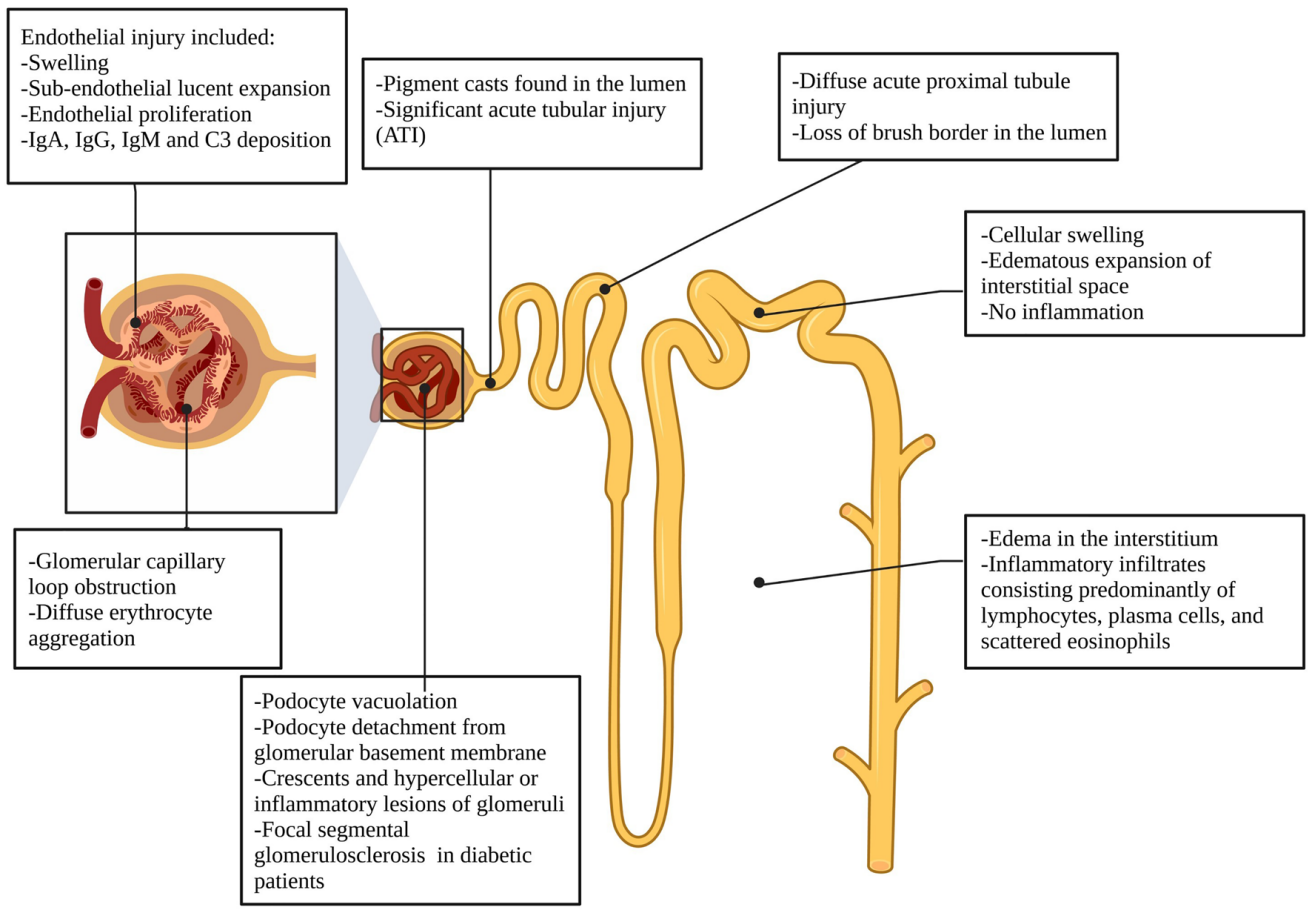
Histopathologic findings in COVID-19 infection. (Created with Biorender.com)

Histological changes were overtly seen in the kidneys of COVID-19 subjects. Although renal parenchyma and the interstitium can be affected, renal biopsy and autopsy records presented significant acute tubular injury (ATI), indicating that interstitial injury is more severe than glomerular damage [68, 77, 78]. The kidney autopsy results showed diffused acute proximal tubular injury with loss of brush border, and pigment casts were observed in the lumen of renal tubules [79]. In addition, glomerular capillary loops were reported to be obstructed, and diffused erythrocyte aggregations were presented [68]. Edema was reported in the interstitium, along with an associated inflammatory infiltrate that predominantly consisted of lymphocytes and plasma cells with scattered eosinophils [71]. Distal tubules and collecting ducts had cellular swelling and edematous expansion of the interstitial space without significant inflammation. The changes with endothelial injury include swelling, sub-endothelial lucent expansion, and endothelial proliferation with the deposition of IgG, IgA, IgM, and C3 [68]. Occasional podocytes vacuolation and detachment from the glomerular basement membrane were also observed. Crescents and hyper-cellular or inflammatory lesions of glomeruli were also noted. Ischemic changes with an accumulation of plasma in Bowman’s space were also documented. Focal segmental glomerulosclerosis (FSGS) was observed in patients with diabetes. Lymphocytes were not presented in glomeruli, and no immune reactants were detected there as well, which suggested that lymphocyte infiltration and immune reactions are uncommon in glomeruli after viral infection [77]. Histopathologic findings in COVID-19 infection are summarized in Fig. 2.

#### The heart–kidney crosstalk in COVID-19 patients

Cardiovascular manifestations, a hallmark of the initial stages of COVID-19 infection, contribute to the development of heart failure, myocardial infarction, and myocarditis through imbalances in the RAAS linked to increased troponin and natriuretic peptides levels. RAAS imbalance further exacerbates the clinical course of infection via microvascular damage, hyperinflammation, and endothelial dysfunction [80]. This inflammatory state may lead to thrombosis and diffuse microangiopathy, or arrhythmias, myocarditis, acute coronary syndrome, and even sudden death [81]. In severe COVID-19 infections, myocardial dysfunction may result from hypoxia, thrombosis, cytokine storm, and/or direct viral injury [82]. Similarly, the hypercoagulable state in critical COVID-19 patients may prompt irreversible kidney damage by extension of the acute tubular necrosis to cortical necrosis, thus causing microinfarctions in the kidney [29]. Further increased circuit clotting has been noted in COVID-19 patients undergoing dialysis [83]. These findings suggest a connection between the renal and cardiovascular systems, which can be explained by the expression of the ACE2 receptor, the COVID-19 port of entry to the cell, in both cardiac and renal tissues. Of note, the expression of ACE2 is downregulated during the course of the infection, thus causing the loss of angiotensin-(1–7) cardio-protection and/or increased action of angiotensin II [84], which could reveal detrimental on the heart and the kidneys.

Reportedly, hypertension coexists along with other cardiovascular disorders in COVID-19 patients [85]. It is significantly prevalent in severe forms of the disease compared to mild cases [86, 87], yet, based on the Centers for Disease Control and Prevention (CDC), hypertension is not considered as a risk determinant of COVID-19 severity [88]. However, chronic kidney disease (CKD) is the commonest indicator of secondary hypertension and an independent contributor to cardiovascular mortality and morbidity [89–91]. Therefore, additional research is needed to unveil the interplay of these variables in the context of COVID-19 infection. In fact, the heart–kidney crosstalk has been strongly implicated by Pelayo et al. (2020), suggesting an unwavering link between acute kidney injury (AKI) and heart failure (HF) in COVID-19 patients. The incidence of HF in AKI patients was nearly four times (19% vs. 4%) more prevalent than the general population of COVID-19-infected patients with increased hypertension (80 vs. 64%) and CKD (27 vs. 9%), thus hinting at the contribution of cardiorenal communication to kidney and heart function deterioration [85]. The cardiorenal syndrome (CRS) may develop in COVID-19 patients with underlined HF [92, 93]. CRS implies that dysfunction in either the heart or the kidney will reciprocally impair the other organ [94]. For example, concomitant COVID-19-induced myocarditis and right-ventricular dysfunction cause venous congestion and diastolic dysfunction with declined cardiac output. The resultant compromised end-organ perfusion similarly translates to kidney injury by jeopardizing its perfusion and creating local congestions [29, 94]. Interestingly, right-ventricular dilation has been strongly linked to an increased risk of death in severe COVID-19 patients [82]. Besides right-ventricular failure, left ventricular dysfunction is directly linked to decreased cardiac output and incomplete arterial filling. Subsequently, the kidneys suffer from hypoperfusion which translates into a reduced glomerular filtration rate (GFR) [95, 96]. In the same vein, cardiomyopathy due to cytokine storm and/or myocarditis can lead to Type 1 CRS phenotype [92, 93] displayed by endothelial damage, which causes intra-abdominal hypertension, edema, pleural effusion, intravascular fluid drop, and hypotension [16, 93]. While Type 3 CRS phenotype occurs as a consequence of cardiomyocyte damage from AKI, Type 5 CRS phenotype is restricted to kidneys and heart damage due to microthrombi, inflammatory response, and increased vascular permeability [16, 92, 93].

From the therapeutic point of view, extracorporeal membrane oxygenation (ECMO) is widely used in COVID-19 patients to sustain the heart and lungs, sometimes coupled with continuous kidney replacement therapy (CKRT) [93]. Nonetheless, excessive positive pressure ventilation administered to COVID-19 patients potentially leads to adverse hemodynamic effects of decreased cardiac output, thus amplifying kidney hypoperfusion. Consequently, AKI may be either induced or intensified in COVID-19 subjects, depending on the severity of the case and the implemented therapies [97]. Recommendations for treatment of cardiorenal syndrome in COVID-19 patients suggest maintenance of a mean arterial pressure > 65 mmHg, good oxygenation, and prevention of volume overload. Despite the initial controversy against the use of ACE inhibitors and angiotensin II receptor blockers since they might increase ACE2 expression, RAAS blockade is still recommended as no evidence indicates any deleterious effects [98, 99].

#### Effect of various treatments adopted for COVID-19 on kidney function

COVID-19 may have a biphasic clinical course, with an initial viral replication phase followed by a cascade of inflammatory events. As such, the consensus is generally moving towards the need for a biphasic pharmacological approach for treatment. The first phase of the disease (from the onset of the symptoms up to 7–10 days) is characterized by viral-induced cytopathic effects, and antiviral drugs may be administered (e.g., chloroquine, hydroxychloroquine, lopinavir/ritonavir, and darunavir/cobicistat). The second phase (beginning 7–10 days from the onset of symptoms) is associated with hyperinflammatory and cytokines release syndromes and carries the risk of death. It is characterized by progressive lung involvement and occasionally signs of hemophagocytic syndrome, with escalating needs for oxygen supplementation and ventilatory support. Immunosuppressive and immunomodulatory drugs may be of benefit at this stage (e.g., glucocorticoids, anti-cytokine drugs, tocilizumab) [100]. In severe cases, hypercoagulability states are reached in response to the cytokine storms, and anticoagulation therapies are imperative to avoid various organ failure and death (unfractionated heparin enoxaparin and low-molecular-weight heparins) [101].

For patients with kidney disease, the management of COVID-19 poses a great challenge, especially in those who are immunosuppressed or with severe comorbidities [100]. Given the high incidence of kidney involvement in COVID-19 and the increased mortality in patients with pre-existing chronic kidney disease (CKD) and those undergoing renal replacement therapy with hemodialysis, it is essential to thoroughly examine all available treatment options and consider their effects on renal function [96]. Furthermore, to reduce the incidence and severity of acute kidney disease in critically ill COVID-19 patients with kidney conditions, it might be useful to implement the Kidney Disease: Improving Global Outcomes (KDIGO) supportive care guidelines (ex: avoidance of nephrotoxins, regular monitoring of serum creatinine and urine output, consideration of hemodynamic monitoring), though this requires validation [102]. In fact, personalized therapy in COVID-19 ill patients is imperative to avoid treatment-induced kidney failure. Universally adopted treatment for COVID-19 and their kidneys-related complications are detailed below.

## Antivirals

### Azithromycin

Azithromycin (AZM) is a macrolide-type antibiotic used mainly to treat respiratory infections. It has shown broad antiviral effects at both in vitro and in vivo stages against Ebola, Zika, influenza H1N1, respiratory syncytial virus, and rhinoviruses. In the case of SARS-CoV-2, it has shown synergistic effects when used with chloroquine and hydroxychloroquine in clinical settings [103]. AZM acts by binding to viral particles and blocking their attachment to lipid rafts, while chloroquine competitively prevents virus binding to gangliosides. Their similar mechanisms of action might explain the synergistic effects of the combination therapy [104]; however, combination therapy was associated with serious side effects such as prolonged QT interval and other GI symptoms like nausea, vomiting, and diarrhea, particularly in patients with a GFR less than 10 mL/min [105]. Azithromycin is eliminated mainly in the gastrointestinal tract via the biliary secretion, while liver metabolism renders inactive metabolites eliminated in the urine; thus, no dose adjustments are needed in patients with renal disease [106]. Randomized clinical trials showed no clinical benefit; therefore, the use of azithromycin, alone or in combination with hydroxychloroquine, for treating COVID-19 was dropped [107–110].

### Favipiravir

Favipiravir (FP) [6-fluoro-3-hydroxy-2-pyrazinecarboxamide (T-705, favipiravir)] is an RNA-dependent RNA polymerase (RdRp) inhibitor approved only in Japan for its anti-influenza activity [111]. FP might also work against COVID-19 via inhibiting the RNA polymerase [112]. The FP regimen against COVID-19 in Japan consists of 1600 mg three times daily (TID) on day 1, followed by 600 mg TID for 4 days [113] amidst a lack of published data on the dosage, duration safety, and efficacy of this drug in COVID-19 treatment [113, 114]. FP was also approved in China for use in COVID-19 treatment [115], and preliminary data of a Chinese clinical trial unveiled better antiviral activity than lopinavir/ritonavir [116]. Moreover, FP administration has resulted in a shorter hospital stay and decreased the need for mechanical ventilation [117]. FP is not recommended for the treatment of COVID-19 as current data on FP showed no clear conclusion since most of these studies had other therapies administered along with FP [118–120].

FP is extensively metabolized by the liver to produce inactive oxidative metabolites progressively excreted in the urine, reaching 80–100% in a week [121], thus necessitating proper monitoring of elderly patients and those with kidney malfunction. Nonetheless, no evidence calls for dose supplementation to dialysis treatments [122].

### Remdesivir

Remdesivir and other nucleoside and nucleotide analogs were first used as broad-spectrum antivirals in the treatment of HIV, herpesvirus, and Hepatitis B and C infections. However, they were used more often after showing efficiency against viral families such as *Picornaviridae, Flaviviridae, Caliciviridae*, and *Coronaviridae*, due to amino acid sequence similarities with HCV [123], and phylogenetic similarities to the RNA-dependent polymerases [124]. Remdesivir is converted to an active metabolite, Remdesivir triphosphate, that targets and competitively inhibit the viral RNA genome replication mechanism [125]. In COVID-19, it works to terminate RNA synthesis at three positions after its incorporation into the strand [126]. Remdesivir has been shown to have in vitro activity against SARS-CoV-2 [127].

The US Food and Drug Administration (FDA) has approved Remdesivir, under the brand name Veklury, to be used as an emergency oral treatment in pediatric patients [128]. COVID-19 patients receiving remdesivir showed a faster recovery rate in a 10-day period as compared to patients taking a placebo [129]. The US FDA issued an emergency authorization for the use of baricitinib, a Janus kinase inhibitor, in combination with remdesivir. Baricitinib has been reported to have antiviral effects via interference with the viral entry, in addition to its immunomodulatory effects [130]. In patients with renal impairment, the pharmacokinetics of remdesivir in these patients is unclear; in addition, since it is prepared in a cyclodextrin vehicle, it can accumulate to toxic levels in patients with renal impairment. However, cyclodextrin vehicle is used in small concentrations, and the treatment duration with remdesivir is relatively short. Thus, the risks of toxicity can be considered minimal [131]. Several studies reported that Remdesivir is safe in patients with renal impairment [132]. The national clinical management protocol stated that Remdesivir is contraindicated in patients with a GFR < 30 mL/min, and in patients on hemodialysis [133, 134]

### Lopinavir/ritonavir

The drug combination “lopinavir-ritonavir” was proposed as an antiviral treatment for COVID-19 [135]. Lopinavir is an HIV-1 protease inhibitor and is combined with ritonavir to increase its plasma half-life [130, 136]. It is indicated for HIV-1 infections in adult and pediatric patients [137]. A study of lopinavir–ritonavir in a ferret model of COVID-19 showed reduced clinical symptoms in treated animals with no effect on viral titers [138]. Results from a randomized clinical trial performed by a Chinese group found no additional benefit of using lopinavir/ritonavir in hospitalized adult patients with severe infection beyond standard care [139]. In fact, The National Institutes of Health’s (NIH) COVID-19 guidelines recommended against the use of lopinavir/ritonavir and other HIV-1 protease inhibitors to treat COVID-19 [140].

This drug is primarily excreted by the fecal route, with renal elimination accounting for less than 2% of the total elimination rate [141]. Therefore, no dose adjustment for lopinavir/ritonavir is needed in patients with kidney injury. However, these drugs are highly bound to plasma proteins and can, therefore, be displaced by other serum protein bound-medications that can increase lopinavir/ritonavir free fractions in these patients [105]. Additional precautions and close monitoring are required in elderly patients since the data on the possible reactions in these patients remain insufficient [142, 143].

## Anti-inflammatory drugs

### Dexamethasone and other glucocorticoids

Glucocorticoids are natural hormones produced by the adrenal cortex. They have potent inflammatory and immunosuppressive properties by inhibiting several intracellular proinflammatory pathways. Glucocorticoids were often used in SARS and MERS outbreaks and are widely used in the COVID-19 pandemic [144]. A daily dose of 6 mg of dexamethasone reduced the monthly mortality rate in COVID-19 patients receiving supplemental oxygen therapy when compared to those with the usual care, and patients in need of mechanical ventilation benefited the most [145]. Dexamethasone (or other glucocorticoids) is not recommended for the treatment in patients with mild-to-moderate COVID-19 [146, 147]. However, according to the World Health Organization, corticosteroids are usually not recommended as they inhibit viral clearance and exacerbate viremia [148]. Moreover, within 24 h of intake, 65% of dexamethasone is excreted unchanged in urine and may cause fluid retention in the elderly or in patients suffering from kidney failure. Dexamethasone dosage is not altered by dialysis, but patients using methylprednisolone should maintain the constant usual dosing procedure after dialysis [149].

### Tocilizumab

Tocilizumab (TOC) is a humanized monoclonal antibody that inhibits the interleukin‐6 (IL-6) receptor to potentially reverse the effects of COVID-19-induced cytokine storm [150, 151]. TOC decreased the mortality rate in severe COVID-19 patients [152] and is reserved for this category of patients [151] due to immunosuppression and increased risk of infection [153]. However, a recent meta-analysis did not reveal a statistically significant rise in infection rates in TOC-administered patients compared to those given the standard of care [152]. Larger randomized clinical trials adjusted for the standard of care medications are needed to establish guidelines for TOC administration in severe cases [152]. No TOC dose adjustments are recommended in patients on dialysis [149]. Tocilizumab is not renally eliminated due to its high molecular weight (148 kDa) [154] and limited data are available on the effects of TOC on the kidney and its renal clearance [155].

### Unfractionated heparin

Unfractionated heparin is an intravenous anticoagulant drug [156] that exerts its effect by binding and stabilizing the complex formed between antithrombin (AT)-thrombin, and AT-factor Xa, in addition to the inhibition of other clotting factors. This drug is indicated for the prevention and treatment of venous thrombosis and pulmonary embolism (PE), prevention of mural thrombosis after myocardial infarction (MI), and treatment of unstable angina and MI [157]. Heparin is cleared from the circulation mainly through the liver, and reticuloendothelial cell-mediated uptake into the extravascular space [156].

The observation of high rates of coagulopathy, thrombosis, and venous thromboembolism in COVID-19 suggested that administration of heparin may improve health-related outcomes in those patients [158]. Furthermore, heparin may decrease infectivity by binding to the SARS-CoV-2 spike protein and functioning as a potential competitive inhibitor for viral entry [159]. In patients with kidney failure, estimation of renal function is necessary when prescribing unfractionated heparin due to the increased risk of both thrombotic and bleeding complications. Unfractionated heparin generally does not require dose adjustment with renal dysfunction. However, close monitoring of anticoagulation therapy is recommended when high doses are administered in patients with chronic renal impairment [160]. Patients may benefit from prophylactic doses of heparin, in addition to therapeutic doses, based on individual risk of thrombosis and coagulopathy [161].

### Low-molecular-weight heparins and enoxaparin

Enoxaparin is a low-molecular-weight Heparin (LMWH) with antithrombotic activity, administered subcutaneously or intravenously for the prevention of deep-vein thrombosis (DVT) in abdominal surgery, hip replacement surgery, knee replacement surgery, or restricted mobility [156]. These drugs provide an advantage over unfractionated heparin, such as better predictable bioavailability and longer half-life, simplified dosing, predictable anticoagulant response, lower risk of heparin-induced thrombocytopenia, and osteoporosis [162]. Nevertheless, LMWHs also pose the disadvantage of accumulating in patients with renal failure and therefore have the potential to produce serious bleeding in these patients [163]. Therefore, enoxaparin requires dose adjustment in patients with renal dysfunction (creatinine clearance < 30 mL/min) [156] due to decreased renal clearance (around 30%) and increased bioavailability. Moreover, a large fraction of the active and inactive metabolites (around 40%) is excreted through the renal route and can be accumulated in these patients. In addition, 80% of the drug is bound to plasma protein and easily displaced in patients with renal failure that might increase drugs’ free fractions in the blood [105]. Accordingly, it is recommended to reduce prophylactic doses to 20–30 mg/day and reduce therapeutic doses by 0.5–1 mg/Kg/day in patients with a GFR < 30 mL/min [164].

### Anakinra

Anakinra is an interleukin-1 (IL-1) receptor antagonist used mainly in the treatment of rheumatoid arthritis to prevent permanent articular damage [165]. It was considered as a potential COVID-19 treatment due to the ability of the virus to induce the production of various cytokines such as IL-1β, IL-6, tumor necrosis factor, and others [12]. Anakinra was proven efficient in reducing mortality rates among COVID-19 patients and decreasing the need for mechanical ventilation in severe cases [166], and it is majorly cleared by the kidney [167]. In patients with normal renal function, the recommended dose is 100 mg daily, however, in patients with renal impairment as measured with a GFR < 30 mL/min, the dose is adjusted to 100 mg on alternating days [149].

## Renal replacement therapy

Renal replacement therapy (RRT) is a method of blood purification using a countercurrent exchange of solutes with a dialysis fluid through a semi-permeable membrane. It is commonly indicated for toxin removal, acid–base or electrolyte abnormalities, and chronic or acute renal failure [168]. During the COVID-19 pandemic, hospitals have seen an increasing need for RRT. In 2020, a metanalysis by Robins-Juarez et al. showed that approximately 5% of all COVID-19 patients required RRT [169]. Interestingly, patients with AKI were most likely to require RRT and less likely to recover kidney function when compared to patients without AKI [170]. Moreover, COVID-19 patients admitted to the ICU showed higher rates for RRT (16.5%) [171], while 20–31% of patients showed indications for RRT [172]. The use of RRT in COVID-19 patients could be challenging since it requires monitoring of the procedure from outside the quarantined area (longer tubing) along with higher dialysate flow and longer time for the daily treatment. In addition, COVID-19 patients requiring RRT should not be transferred to a central dialysis unit but rather should receive RRT in the specialized quarantined area. In addition, it was preferred to keep the number of medical personnel available on the floors to a minimum, with limited entry into quarantine rooms [173].

## Renal transplantation

Although the impact of COVID-19 was studied in patients with kidney transplants, there is limited data on kidney transplantation after, or as treatment of, COVID-19 induced kidney injury [174]. Two recent published cases showed the efficacy of kidney transplantation in patients who recovered from COVID-19 that was proven with at least 2 negative PCR tests and serum IgG titers against SARS-CoV-2. Both the donor and recipient were tested negative (PCR) at the time of transplantation [175]. Recommendations by the National Transplant Organization state that a thorough medical history must always be conducted prior to transplantation, with emphasis on inquiries regarding contact with suspected or confirmed COVID-19 cases. To be eligible for transplantation, a negative PCR result must be provided, while viral antigen tests and serology are not considered as adequate alternatives [174].

### Vaccines against COVID-19

To mitigate the spread of COVID-19 and help return society to normal, multiple COVID-19 vaccines were developed and tested at an unprecedented speed. The efficacy and effects of some of the most widely used vaccines are discussed in this section. As of September 2021, all of them were reviewed by the World Health Organization’s Strategic Advisory Group of Experts on Immunization (SAGE), which has issued interim recommendations for their use.

### mRNA-based vaccines

#### Pfizer-BioNTech (BNT162b2)

The BNT162b2 vaccine is a 2-dose mRNA vaccine developed by Pfizer-BioNTech. It is made with a lipid nanoparticle-formulated, nucleoside-modified RNA that encodes a membrane-anchored SARS-CoV-2 spike protein. A multinational, placebo-controlled, observer-blinded, pivotal efficacy clinical trial performed by Pfizer demonstrated a 95.0% efficacy for this vaccine against COVID-19. Among the 43,252 participants aged 16 years and older, this result was generally consistent across age, sex, race, ethnicity, obesity, and presence of a coexisting condition [176]. In addition, under emergency use authorization, the vaccine continues to be available for individuals 12 years of age and older, for the administration of a third dose in certain immunocompromised people, and for a single booster dose in people 65 and older, 18–64 years of age individuals at high risk of severe COVID-19, and 18–64 years of age individuals with high exposure to SARS-CoV-2 [177]. As of August 2021, this vaccines became the first FDA-approved COVID-19 vaccine for patients 16 years and older.

Possible systemic effects of the vaccine include headache, fatigue, and fever [176]. In keeping with the kidney, cases of minimal change disease [178], acute kidney injury [179], and ANCA-associated vasculitis following vaccination [180] were reported.

### Moderna COVID-19 (mRNA-1273)

Similar to the Pfizer-BioNTech vaccine, the Moderna vaccine is also a lipid nanoparticle-encapsulated mRNA-based vaccine encoding the spike protein of the SARS-CoV-2 virus, and it is administered over 2 doses [181]. Results from the phase 3 randomized, observer-blinded, placebo-controlled clinical trial, conducted in the United States and enrolled 30,420 volunteers aged 18 and above attributed a 94.1% efficacy of the mRNA-1273 vaccine against COVID-19 [181]. The FDA issued an EUA for the emergency use of Moderna COVID-19 Vaccine for the prevention of COVID-19, the authorization of use was for individuals 18 years of age and older.

Systemic side effects of the injection included fever, headache, fatigue, myalgia, arthralgia, nausea or vomiting, and chills [181]. With respect to kidney safety, the vaccine has been associated with minimal change disease [182], de novo vasculitis [183], and ANCA glomerulonephritis [184].

## Vector-based vaccines

### Janssen Ad26.COV2.S

The Ad26.COV2.S, also known as Janssen, or Johnson and Johnson, consists of a vector virus using a double-stranded DNA encoding a SARS-CoV-2 spike glycoprotein variant within a recombinant, replication-incompetent human adenovirus type 26 [185, 186]. The Janssen vaccine received emergency approval from FDA in February 2021 to be used in adults aged at least 18 years old [187]. The vaccine is given as a single dose and was found to protect against symptomatic and asymptomatic COVID-19 patients while also proven efficient against critical and severe COVID-19 illness [188].

Common adverse effects included fever, fatigue, headache, myalgia, and pain at the site of injection [189]. Severe side effects, less frequently encountered, included thrombocytopenia syndrome and Guillain–Barré syndrome [190]. Minimal change disease was reported in a case 1-week post-administration of the Janssen vaccine, with the patient presenting with weight gain, foamy urine, and edema, which then worsened with decreased urine volume [191].

### Oxford/AstraZeneca ChAdOx1 (nCoV-19)

The ChAdOx1 nCoV-19, also known as the Oxford-AstraZeneca vaccine, is a replication-incompetent chimpanzee adenovirus vector containing SARS-CoV-2 structural surface glycoprotein genes [192]. Currently, the vaccine did not receive FDA approval, although many countries have authorized its use within their borders for adults aged at least 18 [193]. Although studies have shown the efficacy of a single dose of the ChAdOx1 nCoV-19 vaccine, recommendations stated that a booster dose of the vaccine could be administered at least 12 weeks after the initial dose [194].

Common adverse effects included fatigue, fever, chills, diarrhea, nausea, injection site pain, and myalgia, while more severe, yet less frequent side effects included thromboembolism and blood clots [195, 196]. There has been a case of minimal change disease and severe acute kidney injury 13 days post-administration of the first vaccine dose. The case, however, did not offer conclusive evidence of a correlation between the ChAdOx1 nCoV-19 vaccine and the development of minimal change disease [197].

## Inactivated viruses

### Sinovac-CoronaVac

CoronaVac (Sinovac Life Sciences, Beijing, China) is a 2-dose β-propiolactone-inactivated virus, an aluminum hydroxide-adjuvanted vaccine developed against COVID-19 [198]. A phase 3 clinical trial run in Brazil showed a 51% efficacy of the vaccine against symptomatic COVID-19 cases [199]. Compared with other COVID-19 vaccines, the occurrence of fever after vaccination was relatively low [200]. The reported adverse side events included pain at the injection site, headache, fatigue, and myalgia [199]. There have been case reports of patients presenting with nephrotic syndrome, and acute kidney injury with rapid deterioration in kidney function [201].

### Sinopharm (BBIBP-CorV)

The Sinopharm, or Vero Cell vaccine, is a 2-dose β-propiolactone-inactivated virus, an aluminum hydroxide-adjuvanted vaccine for the prevention against COVID-19. A multi-country phase 2 clinical trial demonstrated an overall efficacy of 78.1%, which was more or less consistent across subgroups varying in age, sex, and comorbidities. The only exception was individuals with diabetes, for which efficacy dropped to 63.7% [202].

Most adverse side effects associated with vaccination were mild to moderate, including headache and fatigue. However, two serious adverse events have been potentially linked to the vaccine, and these are serious nausea and inflammatory demyelination syndrome/acute disseminated encephalomyelitis [202].

## Conclusion

Based on the available literature, renal manifestations are often seen in COVID-19 patients, and they are associated with increased mortality in subjects admitted to the ICU. It is becoming clear that SARS-CoV-2 particles strike the kidneys in addition to the cytokine storm that perpetuates renal damage in these patients. In the same view, it is evident that some of the currently approved medications to treat COVID-19 patients influenced renal function and must be administered with extreme care. Nonetheless, while some of the adopted vaccines were associated with minimal change disease, none of them was discontinued. Thus, kidney safety in COVID-19 patients remains of utmost concern view the central role that kidneys play in regulating blood pressure and filtering blood from toxic substances. Therefore, constant monitoring of kidneys’ fitness along with cardiac hemodynamics in COVID-19 subjects is imperative to reduce the burden of COVID-19 on human lives. This involves close cardiorenal monitoring to prevent kidney damage in COVID-19 patients.
